# Beyond Notes: Why It Is Time to Abandon an Outdated Documentation Paradigm

**DOI:** 10.2196/24179

**Published:** 2021-04-20

**Authors:** Jackson Steinkamp, Jacob Kantrowitz, Abhinav Sharma, Wasif Bala

**Affiliations:** 1 Department of Medicine University of Pennsylvania Philadelphia, PA United States; 2 Department of Medicine Carney Hospital Boston, MA United States; 3 Department of Family and Community Medicine University of Toronto Toronto, ON Canada; 4 Department of Radiology Emory University Atlanta, GA United States

**Keywords:** electronic medical records, health informatics, information chaos, medical documentation, clinicians, medical notes, electronic medical notes, medical team

## Abstract

Clinicians spend a substantial part of their workday reviewing and writing electronic medical notes. Here we describe how the current, widely accepted paradigm for electronic medical notes represents a poor organizational framework for both the individual clinician and the broader medical team. As described in this viewpoint, the medical chart—including notes, labs, and imaging results—can be reconceptualized as a dynamic, fully collaborative workspace organized by topic rather than time, writer, or data type. This revised framework enables a more accurate and complete assessment of the current state of the patient and easy historical review, saving clinicians substantial time on both data input and retrieval. Collectively, this approach has the potential to improve health care delivery effectiveness and efficiency.

Electronic medical record (EMR) documentation is used for clinical, medicolegal, and billing purposes, as well as research, quality improvement, and population health initiatives. Each of these downstream use cases drives documentation requirements and influences day-to-day efficiency, work satisfaction, and quality of patient care. Poorly designed systems lead to the proliferation of out-of-date or incorrect information [1,2], increased time spent searching the medical chart [3], medical errors [2], and clinician burnout [3-5] while limiting the effective use of EMR data for individual and population-level applications.

Many clinicians are intimately familiar with the pathologies of “information chaos” in electronic patient charts [1]: large volumes of duplicate or *copy-pasted* information, scattered information requiring multiple navigation steps to locate, conflicting information, and outright erroneous information. Well-designed systems and workflows should incentivize data entry, storage, and retrieval behaviors that minimize these forms of information chaos. Conversely, when we see rampant information chaos, we should question and re-evaluate our default assumptions about documentation.

Currently, most medical documentation is organized in bundles or containers called “notes”: documents containing many distinct but loosely related clinical observations, interpretations, and plans. Although notes lump together varied data from different sources regarding different topics, they are generally stored as indivisible units, written largely by a single author, and are rarely edited after initial creation. In their current form, notes organize information by time, by clinical thread (subject matter or team ownership; eg, primary team vs consultant), and by responsibility (the writer of a note simultaneously attests to the truth of, and takes responsibility for, all assertions within it). These are the specific organizational principles and design choices that form the *note* paradigm. These principles—and their consequences for documentation—are easy to overlook when the note paradigm is the only paradigm most clinicians know. Notes have been the predominant organizational principle since the era of paper records, but what if *notes* are the wrong organizational paradigm and are largely responsible for the information chaos that plagues modern electronic charts?

Consider a set of notes written by a single clinician over time. Since each note is a disjointed bundle of information, recorded and accessed *separately*, there are two ways for a clinician to treat a set of sequential notes. The first is to treat each note as a complete *state of the patient* at a particular time. This approach has at least one major problem: the vast majority of a patient’s medical information remains the same from time t to time t + 1. With this approach, clinicians not only habitually document the new updates but also copy and paste large portions of unchanged information from one note to the next to keep all of the information about the patient’s current state in one place. This habit contributes to charts overloaded with duplicate information, makes it difficult for a later reader to easily identify what has changed from time t to t + 1, and makes it onerous to truly expunge errors from the chart because errors now require correction in multiple locations. On the other hand, some clinicians use each note to record only the *new updates* in the patient’s state from t to t + 1. This practice scatters data that should be stored in a single place across multiple notes (eg, the time course of a patient’s chronic medical problems), making it difficult for later readers to find relevant information and to piece together complicated medical histories. So, in the note’s organizational paradigm, clinicians are in a lose-lose situation—forced to choose between large-scale information duplication (if each note is treated as a complete *state of the patient*) or information scatter (if each note is treated as a *bundle of updates*). Most clinicians choose not to adhere strictly to either of these strategies, leading to charts bloated with duplicate and yet seemingly incomplete data, medical histories that are onerous to synthesize, and hard-to-correct errors.

Notes are a poor organizational framework for the individual clinician, but they may be even worse for a collaborative medical team. Although little of a patient’s medical information changes depending on the team or physician viewing it, different teams habitually redocument the same information (eg, the history of present illness) in separate notes, representing another large source of duplicated information. When information does differ between teams (eg, differing physical exam findings or a more in-depth cardiology history), it can only be identified by navigating between separate notes. Such practices not only contribute to information scatter and overload but also waste time on duplicate effort and limit optimal collaborative potential within the EMR.

The current note paradigm thus is a key source of information chaos and associated frustrations. We suggest the value of moving toward a nonnote paradigm in a wholesale effort to reimagine what the electronic medical chart should be. First, we suggest organizing information in the chart primarily by topic rather than by time, team, writer, or data type. This means rethinking how information is stored in the chart—not only the narrative text but also the structured data, including orders, medications, laboratory values, imaging, and other diagnostic results. These data should be tightly coupled to the relevant medical problems and associated free-text data, and update in place dynamically.

Next, the narrative medical chart should be reconceived as a dynamic living workspace—a set of shared and editable topics that contain only the most up-to-date information, rather than a set of separate, fixed time slices. Instead of requiring clinicians to painstakingly identify the information in the medical chart that is true at a given time, this approach ensures that the information present at a glance accurately depicts the most current state of the patient by default. However, the past patient states would still be easily accessible, by navigating forward and back through time, with a version history feature or *track changes* functionality (similar to modern word processing software). This feature would enable readers to quickly identify updates to the chart from t to t + 1 or to follow individual medical problems over time at a more granular level than allowed by notes. When documenting, clinicians would update only the changed information and attest to, but not redocument, the unchanged information. This would facilitate granular clinical updates as they occur (eg, a single medication change or a new symptom) without requiring an entirely new note or addendum. This dynamic workspace paradigm would save substantial time on both the data input and retrieval sides while mitigating incentives for information scatter and copy-paste. Problem-based charting is an appropriate but insufficient step toward this ideal.

A nonnote paradigm would also reduce information chaos at the team level. The chart could be reconceived as a fully collaborative workspace where information is, by default, shared among clinicians. Attestation would replace redocumentation as the primary mode of agreement, even across different clinical services. This is something already done in parts of the chart, for instance to confirm the allergies or family history. Disagreements could easily be documented under this system, but in the same topic-defined place, so that future readers can easily see where opinions differ. Customizable views of certain *slices* of information, depending on a user’s needs, could facilitate service- or individual–specific dashboards for particular workflows. The version history system would enable granular tracking of which clinicians made which changes to a given chart, allowing for appropriate assignment of responsibility (for clinical, medicolegal, or billing purposes). Such a system could be applied to reduce duplication and scatter within a single hospital stay (consultants collaborating with primary teams) and across clinical contexts (an inpatient clinician collaborating with an outpatient clinician). Under such a system, to copy and paste text from another person’s *note* would seem absurd—after all, the two clinicians share and edit the same dynamic workspace.

Deeper than specific software interfaces or billing requirements, our reliance on the note as the primary mode of information organization is a major contributor to clinicians’ EMR frustrations. The note is largely a historical relic from the era of physical paper records, which has outlived its usefulness in the digital era. By questioning historical assumptions and developing alternative documentation paradigms, we can reduce information chaos, promoting improved clinical care, efficiency, and health care worker satisfaction.

## Abbreviations

EMR: electronic medical record
